# A bioprocess perspective on the production of secondary metabolites by *Streptomyces* in submerged co-cultures

**DOI:** 10.1007/s11274-021-03141-z

**Published:** 2021-09-07

**Authors:** Tomasz Boruta

**Affiliations:** Department of Bioprocess Engineering, Faculty of Process and Environmental Engineering, Lodz University of Technology, ul. Wolczanska 213, 90-924 Lodz, Poland

**Keywords:** Co-culture, Secondary metabolites, *Streptomyces*, Submerged co-cultivation

## Abstract

Filamentous microorganisms are potent sources of bioactive secondary metabolites, the molecules formed in response to complex environmental signals. The chemical diversity encoded in microbial genomes is only partially revealed by following the standard microbiological approaches. Mimicking the natural stimuli through laboratory co-cultivation is one of the most effective methods of awakening the formation of high-value metabolic products. Whereas the biosynthetic outcomes of co-cultures are reviewed extensively, the bioprocess aspects of such efforts are often overlooked. The aim of the present review is to discuss the submerged co-cultivation strategies used for triggering and enhancing secondary metabolites production in *Streptomyces*, a heavily investigated bacterial genus exhibiting an impressive repertoire of secondary metabolites, including a vast array of antibiotics. The previously published studies on influencing the biosynthetic capabilities of *Streptomyces* through co-cultivation are comparatively analyzed in the bioprocess perspective, mainly with the focus on the approaches of co-culture initiation, the experimental setup, the design of experimental controls and the ways of influencing the outcomes of co-cultivation processes. These topics are discussed in the general context of secondary metabolites production in submerged microbial co-cultures by referring to the *Streptomyces*-related studies as illustrative examples.

## Introduction

Filamentous microorganisms produce a broad spectrum of structurally diverse secondary metabolites (also termed specialized metabolites or natural products), including polyketides, non-ribosomal peptides and terpenes (Keller 2019). These molecules are not directly involved in the cellular processes associated with growth and energy generation, but rather exhibit various ecological functions allowing the producer to thrive in its environmental niche. In the biotechnological context, the bioactivity exhibited by secondary metabolites is of great interest, as they may serve as promising drug leads (Scherlach and Hertweck 2021). The industrial developments in the field of secondary metabolites production were sparked by the discovery and large-scale manufacturing of penicillin, an antibiotic of fungal origin (Fleming 1929; Barreiro and García-Estrada 2019). Since then, great progress has been made not only in terms of the submerged production of secondary metabolites in stirred tank bioreactors but also with respect to screening, isolation and characterization of novel natural products, including the ones that display antimicrobial activity. So far, approximately 23,000 antibiotics have been discovered from microorganisms, including 10,000 molecules contributed by actinobacteria. In this vast group, about 7600 substances were derived from the genus of *Streptomyces* (Salwan and Sharma 2020), an “undisputed champion” among the microbial producers of antibiotics. Considering the rich catalog of isolated natural products, *Streptomyces* can be regarded as a model microbial genus in the field of secondary metabolism research. Importantly, the analysis of sequenced genomes indicates that the biosynthetic repertoire of *Streptomyces* is far from being fully uncovered (Lee et al. 2020). A plethora of molecules still await discovery, some of which could potentially be applied for developing new drugs to be used in the fight against the global threat of antimicrobial resistance (Udaondo and Matilla 2020). However, the complete catalog of secondary metabolites produced by microorganisms is never fully revealed under standard laboratory conditions of single-species cultivation. The non-standard experimental techniques are required to induce the silent biosynthetic pathways and awake the production of cryptic metabolites (Lee et al. 2021). One of the most promising methods is to mimic the naturally occurring interplay between different microbial species by using the co-cultivation approach. The signals provided by an accompanying species in co-culture can act as a key to unlock the treasure trove of secondary metabolites that are never detected in standard monocultures (the monocultures are often referred to as the axenic cultures). Moreover, the co-cultivation approach may in some cases result in the enhanced levels of target products. Inducing and stimulating the production of secondary metabolites in microbial co-cultures is a topic that has been thoroughly reviewed (Arora et al. 2020; Bertrand et al. 2014; Chen et al. 2020; Liu and Kakeya 2020; Zhuang and Zhang 2021), also in the context of the *Streptomyces* genus (Hoshino et al. 2019; Kim et al. 2021). However, the previous reviews were rather focused on the chemical spectrum of newly discovered or enhanced metabolites, not on the bioprocess-related aspects of co-cultivation. This topic should not be overlooked, as the biosynthesis of microbial natural products is known to be dependent on the composition of growth medium, process scale, pH levels, redox status, temperature, reactive oxygen species, biofilm formation, light intensity, incubation period and stress (Brakhage 2013; Frisvad 2012). In principle, having the production of a given secondary metabolite blocked due to certain process-related issues (e.g., the availability of nutrient sources at inhibitory concentrations or the unfavorable pH values) is possible even in the presence of the stimulatory effects exerted by the partner strain. Hence, the biosynthesis of a given metabolite in the medium represents the net effect of numerous factors acting upon the producing cells.

The aim of the present review is to discuss the bioprocess-related aspects of submerged co-cultivation that facilitate or enhance the production of secondary metabolites in *Streptomyces*. The topics include the co-culture initiation strategies, experimental setup, medium composition, process conditions and the challenges associated with designing the experimental controls. The mechanisms of secondary metabolites biosynthesis in co-cultures are also briefly addressed. While *Streptomyces* was chosen as a model genus to discuss the state-of-the-art in the topic of secondary metabolites production in submerged microbial co-cultures, many of the points discussed here remain valid also in the context of other filamentous microorganisms.

## Methodological challenges of co-cultivation

From a historical perspective, the studies on the enhancement of secondary metabolites production in conventional submerged monocultures of filamentous microorganisms are of fundamental importance for industrial-scale biotechnological developments. Rooted in the times when the submerged production of penicillin was first introduced, the efforts aimed at improving the yields, titers and productivities of target secondary metabolites have been conducted for several decades and involve many aspects of bioprocess optimization, e.g., medium development, aeration and agitation characteristics, cultivation mode (batch, continuous or fed-batch), inoculum propagation, etc. (Calam and Ismail 1980; Mandenius and Brundin 2008). When dealing with a single microbial strain, the performance of the production process depends on the intrinsic properties of the producer itself and the imposed cultivation conditions. When a second strain is involved, the complexity of the system increases considerably as the catabolic and biosynthetic characteristics of both partners, as well as the plethora of chemical and physical interactions between them, must be accounted for. Typically, the two microorganisms have distinct roles in the metabolites-producing “microbial team”, i.e., the producer serves as the cell factory which is stimulated by the accompanying strain to produce the target molecule. Similarly as in the case of axenic cultures, the performance of the process depends greatly on the biomass build-up of the producer as the prerequisite for reaching reasonable productivity levels (Liang et al. 2020). If the growth of the producer is suppressed by the strain that should serve as the stimulating factor, the production-related outcomes are far from satisfactory. This is commonly observed in the preliminary experimental phase before the effective co-culture protocol is devised. On the other hand, if the growth of the stimulating microorganism is practically non-existent, the differences between the mono- and co-cultures may be barely visible, and the very process of co-cultivation becomes unjustified. The experimental challenge is to find a trade-off between maintaining the stimulatory activity and avoiding the suppression of the producing microorganism (Carlson et al. 2015; Mavituna et al. 2016). Even though the understanding of microbial interactions is in most cases still insufficient to provide solid fundaments for rational and prediction-based planning of co-cultivation runs, the straightforward bioprocess-related measures can be taken to shape the outcomes of co-cultures. The key aspects of co-cultivation methodology that should be considered in the experimental design are:

1. the approach of co-culture initiation,
2. experimental setup,
3. co-cultivation medium and process conditions.

All these points are discussed in detail in the following sections.

## The approach of co-culture initiation

Detailed planning of co-culture initiation is of fundamental importance in the design of co-cultivation runs. Many “when and how?” questions need to be answered prior to the start of the process. The experimental approaches suggested in the previously published reports are quite diverse with respect to:

1. the relative quantities of the participating microorganisms,
2. the developmental stage of each microbial partner (vegetative cells or spores),
3. the age (or growth phase) of the precultures used for inoculation,
4. starting the co-culture by inoculating sterile medium or through combining the precultures at the specified proportions,
5. the method of delivering the strains into the co-culture system.

Adjusting the relative quantities of the participating microorganisms is the most common strategy of shaping the co-culture outcomes and avoiding the suppression of the producer by its microbial partner. Practically, this is usually achieved by the modification of inoculation volumes. In their work on undecylprodigiosin production by *Streptomyces coelicolor*, Luti and Mavituna (2011b) mentioned the preliminary experiments towards determining a minimum concentration of *E. coli* cells that would stimulate *S. coelicolor* without overtaking its growth. It was also pointed out in their later effort (Mavituna et al. 2016) that using greater numbers of *E. coli* cells visibly suppressed the proliferation of *S. coelicolor* biomass. Ezaki et al. (1992) emphasized the fact that for the considerable accumulation of biphenomycin A the growth of the producing strain *Pseudomonas maltophilia* 1928 must not be inhibited by the accompanying microbe. To investigate this issue, the authors added the seed culture of *Pseudomonas maltophilia* 1928 to the culture of the producer strain *Streptomyces griseorubiginosus* 43708 at various volume proportions ranging from 0 to 10%. It was determined that the best results in terms of biphenomycin A production were recorded for the 2% ratio (Ezaki et al. 1992). A similar approach was described by Shin et al. (2018), who explored the production of dentigerumycin E by *Streptomyces* sp. JB5 in submerged co-cultures with *Bacillus* sp. GN1. Four different inoculum volume ratios (1:1, 2:1, 5:1 and 10:1) were evaluated and the *Streptomyces*/*Bacillus* ratio of 10:1 turned out to be the most effective option (Shin et al. 2018). Inoculation volume adjustment was also described by Carlson et al. (2015) in the work focused on resistomycin production by *Streptomyces* sp. (strain B033) co-cultured with pathogenic strains of *Proteobacteria*. As reported by the authors, the *Proteobacteria* exhibited relatively fast growth compared to the producer strain and therefore the corresponding volumes had to be optimized accordingly (Carlson et al. 2015). In a different study, Slattery et al. (2001) investigated the enhanced istamycins production in co-cultures and evaluated three different inoculation scenarios, namely (1) the inoculation of the producer (*Streptomyces tenjimariensis*) 24 h before the competitor (a marine bacterial isolate), (2) the inoculation of the competitor 24 h before the producer or (3) the simultaneous inoculation of both strains. According to the authors, in order to control for the "head start" provided to one of the microorganisms under scenarios 1 and 2 the cells were counted after 24 h of growth, and the inoculation of the microbial partner was performed at cell concentrations equivalent to those observed in the pre-established cultures (Slattery et al. 2001). When investigating a large number of co-cultivation variants involving various strains, it is sometimes necessary to modify the existing protocol and make certain exceptions with respect to the designed inoculation scheme. An example was provided in a recent study of Liang et al. (2020), who comparatively evaluated the outcomes of co-cultivation in comparison with the induction by heat-killed cells. Typically, these authors added 100 μL of partner strain seed cultures to the 72-h culture of the producer. However, when *Mycobacterium smegmatis* ATCC 12051 was applied as an inducing strain the inoculation volume was increased to 1 ml in order to achieve comparable cell densities. This strategy led to the successful up-regulation of carbazoquinocin G biosynthesis in *Streptomyces* sp. RKND-216 (Liang et al. 2020). To sum up, two distinct approaches of inoculum adjustments can be listed, namely optimizing the initial cell number ratios towards achieving satisfactory production-related performance or equalizing the biomass levels of the two participating microorganisms at the time of co-cultivation start.

If the experimental approach assumes the use of sporulating microorganisms, the decision regarding the developmental stage is required. In the case of actinobacteria and filamentous fungi, it is the choice between the vegetative cells and spores. Practically, the preparation of the vegetative cells is associated with performing the submerged preculture (seed culture) from which the specified volumes are drawn to inoculate the sterile production medium or to be combined with the preculture of the partner strain. The decision to employ the spores or the preliminarily developed biomass in the form of preculture can be analyzed mainly in the context of the possible morphological scenarios. For example, let us consider mixing the spores of two pellet-forming species of *Streptomyces* that differ markedly in terms of the exhibited growth rates. The fast-growing microbe germinates and develops the pelleted structures before its partner, possibly “sweeping” the spores that have not yet germinated and entrapping them within the pellet cores (Boruta et al. 2019). Hence, the slow-growing microbe is not able to develop its pellets as it typically would in a conventional monoculture. This, in turn, directly affects the spectrum of interactions that may be established in the co-culture. Now, if the same two species are inoculated at the preculture stage, the “pellet versus pellet” scenario takes place, and the interactions involve direct physical contact between the filamentous cells. Other scenarios are also possible, depending on the relative growth rates of both microorganisms and the specific characteristics of the investigated microbial system. Importantly, the “spores versus spores” and “preculture versus preculture” approaches should be regarded as complementary, as they may result in markedly different (but equally interesting) behaviors in terms of secondary metabolites production. The repertoire of options is even broader if the “preculture versus spores” approaches are also considered. Combining the microorganisms at different developmental stages (i.e., strain “A” as spores and strain “B” as vegetative cells) may be considered whenever there is a drastic difference in growth rates between the partners, and providing a growth-related advantage is intended to “help” the slow-growing microbe. While the co-culture initiation tests involving various developmental stages of filamentous fungi can be found in the literature (Boruta et al. 2019), the investigation of this kind is yet to be performed for the *Streptomyces*-derived secondary metabolites. So far, the investigations aimed at inducing or enhancing the production of secondary metabolites in *Streptomyces* relied mainly on the “preculture versus preculture” approach (Table 1). The exception was the effort described by Meschke et al. (2012), who initiated the co-cultures of *Streptomyces lividans* with *Verticillium dahlia* by using the spores of both organisms and observed the stimulated production of streptorubin B and undecylprodigiosin compared to the monoculture variants.

**Table 1:** The bioprocess-related summary of the studies reporting the induction or enhancement of secondary metabolites production by various species representing the *Streptomyces* genus in submerged co-cultures

References	Products	Was the production in co-culture induced (awaken) or enhanced compared to monoculture?	Producer	Partner	Medium components	Initial pH	Co-cultivation vessel (flasks, bioreactors, plates, etc.)	Co-cultivation conditions	The developmental stage at the time of co-culture start	Co-inoculation of sterile medium or combination of precultures?	How were the cells delivered to the co-culture system?
Carlson et al. (2015)	Resistomycin	Induced	*Streptomyces* sp. (strain B033)	Selected *Proteobacteria*	Starch, yeast extract, peptone, filtered lake water or deionized water or the solution of Instant Ocean in deionized water	Not specified	250-mL Erlenmeyer flasks	Shaking at 220 rpm; temperature 20 °C	Vegetative cells	Combination of precultures	In preculture broth
Cho and Kim (2012)	Lobocompactol	Enhanced (10.4-fold increase in concentration)	*Streptomyces cinnabarinus* PK209	*Alteromonas* sp. KNS-16	Tryptone, casitone, glucose, Fe_2_(SO_4_)_3_·4H_2_O, KBr, seawater	7.8	2.8-L Fernbach flasks	Shaking at 215 rpm; temperature 25 °C	Vegetative cells	Combination of precultures	In preculture broth
Ezaki et al. (1992)	Biphenomycin A	Enhanced (60-fold increase in concentration)	*Streptomyces griseorubiginosus* 43708	*Pseudomonas maltophilia* 1928	Soluble starch, Pharmamedia, dried yeast, gluten meal, MgSO_4_·7H_2_O, KH_2_PO_4_, Na_2_HPO_4_·12H_2_O	6.4	200-ml Erlenmeyer flasks	Shaking at 250 rpm; temperature 30 °C	Vegetative cells	Both methods were tested	In preculture broth
Hoshino et al. (2015a)	Niizalactams A-C	Niizalactams A and B—induced; niizalactam C—enhanced (approx. fivefold increase of metabolite levels based on peak area)	*Streptomyces* sp. NZ-6	*Tsukamurella pulmonis* TP-B0596	Glucose, glycerol, soluble starch, Pharmamedia, yeast extract, HP-20	7.0	500-ml baffled Erlenmeyer flask	Shaking at 160 rpm; temperature 30 °C	Vegetative cells	Co-inoculation of sterile medium	In preculture broth
Hoshino et al. (2015b)	Chojalactones A-C	Induced	*Streptomyces* sp. CJ-5	*Tsukamurella pulmonis* TP-B0596	Glucose, glycerol, soluble starch, Pharmamedia, yeast extract, HP-20	7.0	500-ml baffled Erlenmeyer flask	Shaking at 160 rpm; temperature 30 °C	Vegetative cells	Co-inoculation of sterile medium	In preculture broth
Hoshino et al. (2015c)	Arcyriaflavin A; BE-13793C; arcyriaflavin E	Arcyriaflavin A and E—induced; BE-13793C—enhanced	*Streptomyces cinnamoneus* NBRC 13823	*Tsukamurella pulmonis* TP-B0596	Glucose, glycerol, soluble starch, Pharmamedia, yeast extract, HP-20	7.0	500-ml baffled Erlenmeyer flask	Shaking at 160 rpm; temperature 30 °C	Vegetative cells	Co-inoculation of sterile medium	In preculture broth
Jiang et al. (2021)	Longicatenamides A–D	Induced	*Streptomyces* sp*. KUSC_F0*	*Tsukamurella pulmonis* TP-B0596	Oatmeal, glucose, dextrin, fish meal, molasses, Pharmamedia, ebios, CaCO_3_	Not specified	500-ml Erlenmeyer flask	Shaking at 150 rpm; temperature 28 °C	Vegetative cells	Co-inoculation of sterile medium	In preculture broth
Khalil et al. (2019)	Heronapyrrole B	Induced	*Streptomyces* sp. CMB-StM0423	*Aspergillus* sp. CMB-AsM0423	Starch, yeast extract, peptone, artificial sea salt	Not specified	24-well plate microbioreactor (1.5 ml)	Shaking at 190 rpm; temperature 27 °C	Vegetative cells	Combination of precultures	In preculture broth
Liang et al. (2020)	N-Carbamoyl-2-hydroxy-3-methoxybenzamide	Induced	*Streptomyces* sp. RKND-216	*Alteromonas* sp. RKMC-009	Yeast extract, malt extract, glucose, Instant Ocean	Not specified	25 × 150 mm culture tubes or Fernbach flasks	Shaking at 200 rpm; temperature 30 °C	Vegetative cells	Combination of precultures	In preculture broth
Luti and Mavituna (2011a)	Undecylprodigiosin	Enhanced (2.6-fold increase in concentration)	*Streptomyces coelicolor* A3 (2)	*Bacillus subtilis* ATCC 6633	Glucose, NaNO_3_, NaCl, Na_2_SO_4_, K_2_HPO_4_, TRIS base, MgSO_4_·7H_2_O, ZnSO_4,_ trace elements solution	7.2	500-ml shake flasks	Shaking at 200 rpm; temperature 30 °C	Vegetative cells	Co-inoculation of sterile medium	In preculture broth (producer); in saline solution (partner)
Luti and Mavituna (2011b)	Undecylprodigiosin	Enhanced (sixfold increase in concentration)	*Streptomyces coelicolor* A3 (2)	*E. coli* C600	Glucose, NaNO_3_, NaCl, Na_2_SO_4_, K_2_HPO_4_, TRIS base, MgSO_4_·7H_2_O, ZnSO_4,_ trace elements solution	7.2	2-l bioreactor	Aeration rate 2 l/min; agitation 200 rpm; temperature controlled at 30 °C	Vegetative cells	Both methods were tested	In preculture broth
Maglangit et al. (2020)	BE-13793C	Induced	*Streptomyces* sp. MA37	*Pseudomonas* sp.	Yeast extract, malt extract, glucose	Not specified	Co-culture device with two chambers and membrane filter	Shaking at 180 rpm; temperature 28 °C	Vegetative cells	Co-inoculation of sterile medium	In preculture broth
Mavituna et al. (2016)	Undecylprodigiosin	Enhanced (3.5-fold increase in concentration)	*Streptomyces coelicolor* A3 (2)	*E. coli* C600	Glucose, NaNO_3_, NaCl, Na_2_SO_4_, K_2_HPO_4_, TRIS base, MgSO_4_·7H_2_O, ZnSO_4,_ trace elements solution	7.2	500-ml shake flasks	Shaking at 200 rpm; temperature 30 °C	Vegetative cells	Co-inoculation of sterile medium	In preculture broth (producer); in saline solution (partner)
Meschke et al. (2012)	Streptorubin B and undecylprodigiosin	Enhanced (fourfold increase in undecylprodigiosin levels; 4.3-fold increase in streptorubin B levels based on peak area)	*Streptomyces lividans* 66	*Verticillium dahliae*	L-asparagine, K_2_HPO_4_, MgSO4·7H2O, FeSO_4_·7H_2_O, glucose	Not specified	Flasks	Temperature 25 °C	Spores	Co-inoculation of sterile medium	not specified
Onaka et al. (2011)	Actinorhodin; undecylprodigiosin	Induced	*Streptomyces lividans* TK23	One of mycolic acid-containing bacteria	Glucose, glycerol, soluble starch, Pharmamedia, yeast extract, HP-20	7.0	500-ml K-1 flasks or dialysis flasks with two chambers	Shaking at 200 rpm; temperature 30 °C	Vegetative cells	Co-inoculation of sterile medium	In preculture broth
Onaka et al. (2011)	Alchivemycin A	Induced	*Streptomyces endus* S-522	*Tsukamurella pulmonis* TP-B0596 or *Corynebacterium glutamicum* ATCC13869	Glucose, glycerol, soluble starch, Pharmamedia, yeast extract, HP-20	7.0	500-ml K-1 flasks or dialysis flask with two chambers	Shaking at 200 rpm; temperature 30 °C	Vegetative cells	Co-inoculation of sterile medium	In preculture broth
Park et al. (2017)	Gordonic acid	Induced	*Streptomyces tendae* KMC006	*Gordonia* sp. KMC005	Yeast extract, malt extract	Not specified	Flask	Not specified	Vegetative cells	Co-inoculation of sterile medium	In preculture broth
Pérez et al. (2011)	Actinorhodin	Enhanced (approx. 20-fold increase of metabolite levels based on peak area)	*Streptomyces coelicolor*	*Myxococcus xanthus*	Casitone, MgSO_4_, Tris–HCl, potassium phosphate	7.6	100 ml baffled flasks	Temperature 28 °C	Vegetative cells	Combination of precultures	In preculture broth
Schäberle et al. (2014)	Undecylprodigiosin	Enhanced (60-fold increase in concentration)	*Streptomyces coelicolor* M145	*Corallococcus coralloides* B035	Yeast extract, malt extract, glucose	7.3	300-ml Erlenmeyer flasks	Shaking at 140 rpm; temperature 30 °C	Vegetative cells	Combination of precultures	the use of liquid was not mentioned
Shin et al. (2018)	Dentigerumycin E	Induced	*Streptomyces* sp. JB5	*Bacillus* sp. GN1	Yeast extract, malt extract, glucose, artificial seawater	Not specified	500-mL baffled Erlenmeyer flask	Shaking at 200 rpm; temperature 30 °C	Vegetative cells	Co-inoculation of sterile medium	In preculture broth
Slattery et al. (2001)	Istamycin A and B	Enhanced (approx. twofold increase in concentration)	*Streptomyces tenjimariensis* SS-939	One of 53 marine bacterial isolates	Potato starch, soybean meal, ferric phosphate, seawater	7.6	Shake flasks	Shaking (speed not specified); Temperature 25 ± 1 °C	Vegetative cells	Both methods were tested	In preculture broth
Sugiyama et al. (2015)	5-Alkyl-1,2,3,4-tetrahydroquinolines	Induced	*Streptomyces nigrescens* HEK616	*Tsukamurella pulmonis* TP-B0596 or Corynebacterium glutamicum	Not explicitly given, the general approach of "combined culture" (Onaka et al. 2011) was followed				Vegetative cells	Co-inoculation of sterile medium	In preculture broth
Sugiyama et al. (2016)	Streptoaminals	Enhanced	*Streptomyces nigrescens* HEK616	*Tsukamurella pulmonis* TP-B0596	Not explicitly given, the general approach of "combined culture" (Onaka et al. 2011) was followed				Vegetative cells	Co-inoculation of sterile medium	In preculture broth
Sung et al. (2017)	Granaticin, granatomycin D, and dihydrogranaticin B,	Enhanced (23-, 15- and 26-fold increase in peak area for granatomycin D, granaticin and dihydrogranaticin B, respectively)	*Streptomyces* sp. PTY087I2	Selected human pathogens	Yeast extract, soluble starch, trypticase peptone, Instant Ocean	Not specified	125-ml flask	Shaking at 200 rpm; temperature 30 °C	Vegetative cells	Combination of precultures	In preculture broth
Wakefield et al. (2017)	Chaxapeptin; nocardamine	Chaxapeptin—induced; nocardamine—enhanced	*Streptomyces leeuwenhoekii* C34	*Aspergillus fumigatus* MR2012	Glucose, yeast extract, malt extract	Not specified	Flask	Shaking at 180 rpm; temperature 30 °C	Vegetative cells	Co-inoculation of sterile medium	In preculture broth
Wakefield et al. (2017)	Nocardamine; pentalenic acid, chaxapeptin	Nocardamine; pentalenic acid—induced; chaxapeptin—enhanced (twofold increase of metabolite levels based on peak area)	*Streptomyces leeuwenhoekii* C58	*Aspergillus fumigatus* MR2012	Glucose, yeast extract, malt extract	Not specified	Flask	Shaking at 180 rpm; temperature 30 °C	Vegetative cells	Co-inoculation of sterile medium	In preculture broth
Yu et al. (2019)	Borrelidins J and K	Induced	*Streptomyces rochei* MB037	*Rhinocladiella similis* 35	Malt extract, anhydrous dextrose, yeast extract, artificial seawater	pH 7.0	Not specified	Shaking at 180 rpm; temperature 28 °C	Vegetative cells	Combination of precultures	In preculture broth

If the precultures are chosen to be used for inoculation, the “history” of the inoculum needs to be considered when comparing the results of co-cultivation experiments, because it determines the physiological state of the cells at the time of co-culture start. The preculture is nothing more than a conventional batch monoculture, in which one may distinguish distinct phases of biomass build-up. The most typical, textbook situation for the bacterial cells is to observe the lag phase, the exponential growth phase, the stationary phase and the death (decline) phase. While the exact shape of the growth curve depends on the given microbial system, what matters in the context of co-culture inoculation is that the cells representing different phases differ not only in terms of their growth characteristics, but also exhibit distinct biosynthetic capabilities. So, the age of the preculture determines the chemical repertoire that the strain can display at the time of co-culture start and its readiness for generating a wide array of secondary metabolites to interact with its partner. If the cells enter the co-culture system together with the portion of the broth, it should be remembered that the liquid itself may contain a rich cocktail of metabolites (the older the preculture the greater amounts of these can be expected to accumulate in the preculture broth). The “history” of the seed culture can be considered either by directly referring to the duration of the preculture (e.g., in days or hours) or indirectly by reporting the changes of biomass concentration throughout the preculture period. When using the direct time-based method, it must be remembered that any meaningful comparisons can be made solely if referring to the same microorganism. Due to the physiological differences observed among different species, most importantly with respect to their attainable growth rates, the time necessary to enter the respective growth phases may differ substantially depending on the microorganism. For example, the cells of two distinct species can arrive at the equivalent physiological states at markedly different preculture times. In these cases, reporting the course of biomass concentration or optical density (OD) could be seen as a recommended approach. For example, the OD_600_ values corresponding to the precultures were shared by Sung et al. (2017), who co-cultivated *Streptomyces* sp. PTY087I2 with the selected human pathogens and noted the stimulated production of granaticin, granatomycin D, and dihydrogranaticin B. Most of the reports, however, refer to the duration of the preculture. In some cases, the authors describe the adjustments required to reach the desired physiological states of both precultures at the time of inoculation, e.g., by inoculating the preculture of one strain a day or two before the second strain. This was performed, for example, in the study of Wakefield et al. (2017), where the fungal preculture of *Aspergillus fumigatus* MR2012 was started 2 days before the preculture of *Streptomyces leeuwenhoekii* C58. Such an approach allows the slower-growing partner to develop its biomass and “prepare” for entering the target co-culture system.

The initiation of submerged co-cultures is performed according to two general strategies, namely through the simultaneous inoculation (co-inoculation) of sterile medium (with the use of precultures or spores) or by combining the precultures at the specified proportions. Among the previous studies focused on *Streptomyces* as the microbial producers in co-cultures, the co-inoculation method was employed more often than the combination of precultures (Table 1). A broad range of volume ratios used for the co-inoculation was reported in the literature. This could be attributed to the adjustments of relative biomass concentrations that were assumed in the respective experimental protocols. In some of the works, equal volumes of precultures were used for inoculations. For example, Wakefield et al. (2017) in the study on the production of chaxapeptin, nocardamine and pentalenic acid simultaneously inoculated 4 l of production medium with 200 ml of seed cultures of *Streptomyces leeuwenhoekii* (strain C34 or C58) and *Aspergillus fumigatus* MR2012. In most reports, however, volume ratios other than 1:1 were used. For example, in a series of experiments involving the so-called “combined cultures” of *Streptomyces* with mycolic acid-containing bacteria (Hoshino 2015a, 2015b, 2015c; Jiang et al. 2021; Onaka 2011; Sugiyama et al. 2015, 2016), the authors typically used the volume ratio of 3 ml/1 ml (producer/partner) to inoculate 100 ml of sterile medium. In the studies involving the alternative strategy, namely the combination of precultures, the broad range of volume ratios was also reported, from 1:1 vol/vol (Yu et al. 2019) to 1000:1 vol/vol (Cho and Kim 2012). Technically, the common approach was to transfer an aliquot of partner strain preculture to the flask containing the producer preculture to trigger the co-cultivation process (Carlson et al. 2015; Cho and Kim 2012; Liang 2020; Pérez 2011; Sung et al. 2017). In one study, the cells were separated from the preculture broth before being added to the monoculture of the partner strain (Schäberle et al. 2014).

Finally, the method of transporting the strains into the co-culture system ought to be considered. The main question is whether the spores or vegetative cells to be used for co-culture initiation are suspended in liquid or not. If so, the type of the liquid needs to be specified. As the production of secondary metabolites by *Streptomyces* in submerged co-cultures was triggered with the use of vegetative cells in most studies, it was not surprising that the preculture broth served as the typical “means of transport” in these cases (Table 1). The reports of Luti and Mavituna (2011a) and Mavituna et al. (2016) were exceptional in this context, because they described the use of the stimulating microorganisms (*B. subtilis* and *E. coli*, respectively) after centrifugation, washing and re-suspending of their vegetative cells in saline solution prior to the co-inoculation with the producer (*S. coelicolor*). Alternatively, suspending the cells in sterile medium may also be considered, but such an approach has not been yet evaluated in terms of *Streptomyces*-based production of secondary metabolites. Liang et al. (2020) employed a mixed approach, namely the inducer preculture was added to the producer cultures together with a small portion of fresh sterile medium. Interestingly, in the work of Schäberle et al. (2014) the preculture of *Corallococcus coralloides* B035 was centrifuged, and the pellet of living cells was transferred to the *S. coelicolor* culture, while suspending the cells in any kind of liquid prior to inoculation was not mentioned.

## Experimental setup

Considering the broad set of co-cultivation methods reported across different fields of biotechnology (Goers et al. 2014; Kapoore et al. 2021), it may be at first surprising to realize that nearly all previous co-cultivation studies regarding the production of secondary metabolites by *Streptomyces* were conducted only at relatively small scales (working volume less than 1 l in most cases) and by employing conventional laboratory flasks (with or without baffles) (Table 1). Among these efforts, the work of Luti and Mavituna (2011b) was the only example of using the stirred tank bioreactor system (total volume and working volume of 2 l and 1.5 l, respectively). To gain deeper understanding of the interactions that proved to be stimulatory for actinorhodin and undecylprodigiosin production in *Streptomyces lividans* TK23, Onaka et al. (2011) employed a specialized type of vessel, namely a dialysis flask consisting of two compartments separated by a membrane. The membrane made it impossible for the participating strains to engage in direct physical contact but allowed chemical communication through exchanging small molecules. Since the production was not observed to be induced under these conditions, it became clear that the physical contact was the basis for exhibiting the stimulatory effect. A very similar 2-chamber setup equipped with a semi-permeable membrane was recently developed by Maglangit et al. (2020) who investigated the indole alkaloid production in the co-cultures of *Streptomyces* sp. MA37 and *Pseudomonas* sp. under submerged conditions. In a different study, Liang et al. (2020) employed the 25 × 150 mm culture tubes that enabled higher throughput of the experimental work. A different approach was applied by Khalil et al. (2019), who used a 24-well plate microbioreactor system for the co-cultivation of *Streptomyces* sp. CMB-StM0423 and *Aspergillus* sp. CMB-AsM0423. However, considering the entire set of literature records focused on *Streptomyces* as the producers of secondary metabolites, the experimental protocols on co-cultivation are still rather based on the standard laboratory equipment. In future studies, it would be interesting to compare the biosynthetic outcomes of co-cultivation processes not only performed under different conditions but conducted with the use of various setups and techniques, e.g., encapsulation, spatial separation or microfluidic systems (recently reviewed by Kapoore et al. 2021).

## Co-cultivation medium and process conditions

Generally, the co-cultivation studies that proved to be successful in terms of secondary metabolites production by *Streptomyces* were not accompanied by the reports on extensive medium development or optimization. Apparently, the choice of the medium took place over the course of the preliminary works and was not regarded as one of the most challenging aspects of the work (the previously published media compositions were often used). The experience in performing the standard monocultures could be thus directly applied in the case of co-cultures. It was not surprising that in several previous studies (Table 1) the standard media for *Streptomyces*, e.g., the ISP2 medium composed of glucose, yeast extract and malt extract, were employed without any modifications (Maglangit et al. 2020; Schäberle et al. 2014; Wakefield 2017). An opposite approach was applied by Pérez et al. (2011), who used the CTT liquid medium tailored towards the cultivation of *Myxococcus xanthus*, which in this case was the partner of *S. coelicolor* in co-culture, and still recorded the satisfactory growth and actinorhodin production by *S. coelicolor*. If at least one of the co-cultured partners was of marine origin, the media were modified to meet the requirements of such organisms and included some additional ingredients, e.g., artificial seawater (Shin et al. 2018; Yu et al. 2019) or the commercially available Instant Ocean® salt mixture (Carlson et al. 2015; Liang et al. 2020; Sung et al. 2017). The initial pH values varied from 6.4 (Ezaki et al. 1992) to 7.8 (Cho and Kim 2012) depending on the study and the strain, with most experiments initiated at the pH levels between 7.0 and 7.2. No pH control strategies were employed, the pH was allowed to change freely during the co-cultivation. Importantly, as already mentioned, no previous works concerned the process of adjusting or rigorously optimizing the medium composition to influence the outcome of the co-culture and ultimately achieve a successful co-cultivation system based on *Streptomyces* as a producer.

As far as the process conditions were concerned (Table 1), the cultivation vessels were shaken at a constant rate between 140 (Schäberle et al. 2014) and 250 rpm (Ezaki et al. 1992). The temperature was controlled in the interval from 20 to 30 °C depending on the study, with the latter value being the most used temperature (Table 1). In the bioreactor-based study conducted by Luti and Mavituna (2011b), the aeration rate of 2 l min^−1^, stirring speed of 200 rpm and the temperature of 30 °C were applied. Importantly, only the batch operational mode was used, no portions of fresh medium were added after the start of the process. Generally, the influence of growth conditions on the results of co-cultivation remains an unexplored topic in the context of secondary metabolites production. The studies published so far leave plenty of room for the bioreactor-based tests and bioprocess characterization of the performance of *Streptomyces* in submerged co-cultures.

## Experimental controls

If the experimental goal is to prove the effectiveness of the co-cultivation approach in terms of inducing or enhancing the production of secondary metabolites, there is a need for running the monoculture controls. In practice, it means that the conventional axenic cultures (i.e., inoculated with a single strain) are propagated in parallel with the investigated co-cultivation variants. There are, however, certain issues associated with designing the controls for the production of secondary metabolites in submerged co-cultures that are not discussed in the literature, mainly related to the broth carry-over. As already mentioned in the context of *Streptomyces*-centered studies, the typical approach to initiate the co-cultivation is to use the precultures, i.e., the vegetative cells suspended in the fermentation broth. So, in the case of the co-cultivation variant the producer strain comes into contact not only with the cells but also with the portion of preculture broth of its partner. The liquid that accompanies the partner strain not only contains certain amounts of nutrients that have not been consumed over the course of preculture but can also be enriched with the molecules secreted by the strain itself. Importantly, its exact chemical composition is unknown. If introduced to the co-culture system together with the cells, the broth becomes an additional factor that affects the outcomes of the co-culture compared to the control, both in terms of medium composition and in terms of volume. Depending on the relative amounts of inoculum and the adopted approach, this effect could be regarded as negligible. However, when the two precultures are combined at comparable volumes at the co-cultivation start, the observable effects exerted by the preculture broth on the production of secondary metabolites can be expected to occur. If the co-culture is investigated as a system that allows to reach certain production-related goals not achievable through conventional cultivation, the study may be seen as an evaluation of a successful black-box responsible for elevating the levels of metabolites or generating novel molecules. In this case, the experiment focuses on demonstrating the effectiveness of the system, rather than proving that the observed effects occur exclusively due to the presence of an accompanying microorganism. If, however, the study aims to rigorously quantitate the changes of metabolic levels occurring specifically due to microbial interactions, additional measures must be taken in the experimental design to account for the broth carry-over and volume differences between the tested co-cultures and the corresponding controls. So far, this issue has not been comprehensively addressed, but the procedure of centrifuging, washing and re-suspending the cells in saline described by Luti and Mavituna (2011a) and Mavituna et al. (2016) is a good example of avoiding the carry-over. However, to avoid the differences in terms of volume, the equivalent portion of the liquid used for re-suspending the cells (saline solution, fresh medium or another medium of known composition) should always be added to the monoculture controls of the producer strain. In general, according to a rigorous approach, whenever the cells are added to the co-culture together with a volume of a given liquid, the same volume of the same liquid should be added to the monoculture controls to eliminate the medium-associated effects. This is usually regarded as negligible in current literature and not addressed when describing the experimental controls.

The additional questions arise when a bioreactor system is used for co-cultivation. The bioreactor setup allows to automatically control the levels of pO_2_ (by the adjustment of stirring speed and aeration rate) and pH (by adding acids or bases) throughout the run, what may be of crucial importance for the secondary metabolites production. In the context of experimental controls, the use of automatic control mechanisms must be well thought over, as it may lead to great differences in terms of process conditions between the controls and the co-culture variants. In the controlled system, the values of stirring speed and agitation rate will be adjusted to meet the oxygen demands (as defined by the pO_2_ level set by the user). As the utilization of oxygen in the monoculture can be expected to differ from the one observed in the co-culture, it is also clear that the mechanical stress in the two variants will not be equivalent. In turn, this would affect the morphology and the productivity of the strains. As already discussed, two paths can be taken here. Either the system is evaluated solely with respect to its production-related outcomes or the effects of microbial interactions are determined and rigorously quantitated. In the latter case, having equivalent hydrodynamic conditions in the mono- and co-culture bioreactors is required. While recommended in the methodological sense, such an approach may lead to markedly different results than the automatic control, not necessarily resulting in richer biosynthetic repertoires. Comparing the outcomes of both strategies may bring interesting insights into the influence of process conditions on the performance of submerged co-cultures. Therefore, the two bioreactor cultivation methods ought to be perceived as complementary in this respect.

## Reaching beyond the “one producer + one partner” scheme

The typical co-cultivation experiment aimed at the secondary metabolites production by *Streptomyces* follows the same scheme of having one producer strain accompanied by one stimulating strain. Such an approach allows to adjust the relative growth rates and arrive at satisfactory production levels with relative ease. Moreover, it allows for the elucidation of specific interactions occurring between the two species. As the research on multi-species microbial systems visibly gains momentum in recent years, especially with regard to designing synthetic microbial consortia (Diender et al. 2021; Grandel et al. 2021; Zaramela et al. 2021), it may be expected that the common “one producer + one partner” scheme will be modified in an increasing number of future studies (Shakeri Moghaddam et al. 2021). The use of *Streptomyces* in microbial consortia was previously reported in the context of environmental biotechnology (Fuentes et al. 2014, 2017; Han et al. 2020) and biofuels production (Bhatia et al. 2015). Theoretically, there are two issues to consider if secondary metabolites are to be generated via the multi-species system, namely the overall number of species included in the co-culture, the number of target products and the number of producer strains. If the experimental idea assumes that there is more than a single producer, the main challenge is obviously to adjust the co-culture initiation method, mainly in terms of inoculation ratios. In this case, the challenge is to ensure that all the producers develop in concert and that their growth rates are sufficient to generate reasonable product titers. Additional issues are associated with designing the controls for every producing strain participating in the co-culture, as was previously communicated in the context of fungal co-cultures (Boruta et al. 2020).

## Investigating the mechanisms of secondary metabolites biosynthesis in co-cultures

The discovery of novel secondary metabolites in co-cultures is often followed by investigating the microbial interactions and biosynthetic mechanisms that resulted in the observable induction of metabolic pathways. The mechanistic analysis may be initiated by testing if the inducing activity can be mediated by diffusible molecules or is rather based on the direct cell-to-cell interactions. Additionally, it can be examined whether the activation of biosynthetic routes may be elicited by adding dead cells of a partner strain instead of performing the actual co-cultivation. Such efforts were described by Onaka et al. (2011), who applied the 2-chamber flask system equipped with a membrane to demonstrate that the production of red pigment in *S. lividans* is triggered by the physical contact with the mycolic acid-containing bacteria (MACB). It was shown that the viable cells of MACB entwined tightly with the biomass of *S. lividans*, whereas the killed cells of MACB did not show such behavior and failed to display the inducing activity. Mavituna et al. (2016) used a different approach, namely the cultivation of *S. coelicolor* in the presence of cell-free supernatant of *E. coli*. In this case, the inducing effect was shown to be based on the class of molecules containing 1,2-benzene dicarboxylic acid moiety secreted by *E. coli* into the fermentation broth. Currently, there are rather few comprehensive reports addressing the genetic mechanisms and chemical signals that govern the secondary metabolites production in *Streptomyces* co-cultures. An example of a detailed and insightful study was provided by Khalil et al. (2019), who demonstrated that chemical communication may trigger the defense mechanisms in the interacting microorganisms. Briefly, the bacteriostatic metabolite *cyclo*-(L-Phe-*trans*-4-hydroxy-L-Pro) produced by *Aspergillus* sp. CMB-StM0423 stimulated *Streptomyces* sp. CMB-StM0423 to produce nitric oxide (NO), which in turn activated the biosynthetic gene cluster of a fungistatic known as heronapyrrole B. Importantly, Khalil et al. (2019) showed that the NO-mediated transcriptional activation (NOMETA) may be widespread across the genus of *Streptomyces.* In a recent study, Honma et al. (2021) reported that NO regulates actinorhodin production in *S. coelicolor* via the heme-based DevS/R two-component system. A mechanism of protecting the bacterial community against the viral threat was investigated by Kronheim et al. (2018), who focused on the secondary metabolites produced by *Streptomyces* as chemical weapons against the infecting DNA phages. Remarkably, it turned out that these antiviral metabolites could be used not only by the producing cell but also by the neighboring cells of *Streptomyces* (even if they represented different species). An interesting mechanism of metabolic induction was described by Kurosawa et al. (2008), who discovered rhodostreptomycins production following the horizontal gene transfer from *Streptomyces padanus* to *Rhodococcus fascians*. In a different study, Traxler et al. (2012) elucidated the mechanisms associated with the activation of siderophores production in the interacting actinomycetes *S. coelicolor* M145 and *Amycolatopsis* sp. AA4 in the context of their competition for iron. A more detailed review concerning the mechanisms of metabolic responses in co-cultures was recently presented by Liu and Kakeya (2020).

## Concluding remarks and outlook

So far, the production of secondary metabolites by *Streptomyces* in submerged co-cultures has been studied only at relatively small scales, mostly in shake flasks. The common approach of combining the microorganisms was the inoculation of sterile medium with the use of precultures or the combination of precultures at the specified volume ratios. There is still a need for detailed investigation of bioreactor co-cultures to determine the influence of scale, mode of operation and process conditions on the results of co-cultivation. As the discovery of new secondary metabolites produced by *Streptomyces* continues, the door will open for large-scale comparative studies involving novel submerged co-cultivations methods, reaching beyond the use of conventional laboratory flasks and stirred tank bioreactors. The future bioprocess-related studies will be performed in parallel with the elucidation of molecular mechanisms governing microbial interactions. Furthermore, the comprehensive and rigorous studies regarding the outcomes of various co-culture initiation strategies will be valuable contributions to the field of *Streptomyces*-related research. Finally, the *Streptomyces*-based synthetic microbial consortia aimed at secondary metabolites production are expected to be designed and characterized.
